# Long-term effects of lifetime trauma exposure in a rural community sample

**DOI:** 10.1186/s12889-015-2490-y

**Published:** 2015-11-25

**Authors:** Tonelle E. Handley, Brian J. Kelly, Terry J. Lewin, Clare Coleman, Helen J. Stain, Natasha Weaver, Kerry J. Inder

**Affiliations:** Centre for Rural and Remote Mental Health, University of Newcastle, Level 5 McAuley Building, Callaghan, 2308 NSW Australia; National Drug and Alcohol Research Centre, University of New South Wales, Sydney, NSW Australia; Centre for Translational Neuroscience and Mental Health, University of Newcastle, Newcastle, NSW Australia; Hunter New England Mental Health Services, Newcastle, NSW Australia; Sydney Centre for ATSI Statistics, University of Sydney, Sydney, NSW Australia; School of Medicine, Pharmacy and Health, Durham University, Durham, UK; Research Services, University of Newcastle, Newcastle, NSW Australia; School of Nursing and Midwifery, University of Newcastle, Newcastle, NSW Australia

**Keywords:** Post-traumatic stress disorder, Trauma, Rural mental health, Psychosocial characteristics

## Abstract

**Background:**

This study examines the long-term outcomes of lifetime trauma exposure, including factors that contribute to the development of PTSD, in a sample of rural adults.

**Methods:**

In 623 rural community residents, lifetime trauma exposure, PTSD, other psychiatric disorders and lifetime suicidal ideation were assessed using the World Mental Health Composite International Diagnostic Interview. Logistic regressions were used to examine relationships between potentially traumatic events (PTEs) and lifetime PTSD and other diagnoses.

**Results:**

78.2 % of participants reported at least on PTE. Rates were broadly comparable with Australian national data: the most commonly endorsed events were unexpected death of a loved one (43.7 %); witnessing injury or death (26.3 %); and life-threatening accident (19.3 %). While the mean age of the sample was 55 years, the mean age of first trauma exposure was 19 years. The estimated lifetime rate of PTSD was 16.0 %. Events with the strongest association with PTSD were physical assault and unexpected death of a loved one. Current functioning was lowest among those with current PTSD, with this group reporting elevated psychological distress, higher mental health service use, a greater number of comorbidities, and lower perceived social support. Respondents with a past PTE but no PTSD history were generally similar in terms of their current wellbeing to those with no lifetime PTE.

**Conclusions:**

PTEs may have diverse psychological and social consequences beyond the development of PTSD. Ensuring that adequate support services are available in rural areas, particularly in the period immediately following a PTE, may reduce the long-term impact of traumatic events.

## Background

People residing in rural areas may experience greater exposure to a range of potentially traumatic events (PTEs) at both a personal and community level. Previous research indicates that rural residents are more likely to experience serious accidents (e.g. motor vehicle accidents and occupational injuries), a higher suicide rate, and a higher frequency and severity of accident-related injuries [1]. In addition there is a greater occurrence of adverse community level events, including severe environmental adversity such as drought, floods and bushfires [2]. The compounding effect of multiple trauma exposure at both personal and community levels, along with lower availability of services and rural attitudes of self-reliance [3], also suggest that rural residents may in turn have an increased likelihood of poorer health outcomes in the event of adversity, such as Post-Traumatic Stress Disorder (PTSD) [4]. This has been reflected in previous Canadian research, which shows a higher rate of PSTD in rural than urban regions [5]. Hence, understanding the impacts of PTEs on rural residents is of particular importance. Although there has been some research investigating the patterns of trauma exposures and outcomes in rural populations [1], these have largely focused on personal psychological outcomes alone, with few studies exploring a broad range of personal factors (such as levels of adversity, social networks) and community-level factors, including rural characteristics (such as service availability and remoteness) on psychological and social outcomes.

Since evidence suggests that some PTEs may occur more frequently in rural regions, knowledge of the long-term impacts of trauma, including factors that promote psychological recovery or mediate risk to the development of psychiatric sequelae such as PTSD, is critical. This analysis examines the lifetime exposure to PTE in a rural adult population, and examines the factors associated with the presence or absence of lifetime PTSD diagnosis using structured diagnostic interview methodology. The study examines the impact of prior PTE with or without PTSD on current psychological and social functioning. We hypothesise that those with a lifetime history of at least one PTE will report poorer current functioning than those with no PTE history, and that this will be particularly marked for those with current PTSD symptoms.

## Methods

### *Participants*

Data were obtained from the 2007–2009 baseline phase of the Australian Rural Mental Health Study (ARMHS), a longitudinal population study of mental health in rural and remote communities that has been described in detail previously [6]. The participants included in the current analysis comprised a stratified subsample (*N* = 623) who completed the PTSD section of the World Mental Health Composite International Diagnostic Interview version 3.0 (WMH-CIDI-3.0) [7], selected according to their psychological distress scores (see below). ARMHS was approved by the Human Research Ethics Committees of the Universities of Newcastle and Sydney, and the Greater Western, Hunter New England and North Coast Area Health Services. All participants provided written informed consent at the time of the postal survey, and re-confirmed their consent orally over the telephone before completing the WMH-CIDI-3.0.

### *Measures*

#### Lifetime trauma exposure, PTSD and other psychiatric disorders

Lifetime PTEs, lifetime and 12-month PTSD diagnosis were examined using the WMH-CIDI-3.0; for the present analyses, ICD-10 diagnoses are reported. A two-stage assessment procedure was undertaken. After completing the K10 psychological distress scale [8] participants were selected for WMH-CIDI-3.0 interview based on their distress score. Interviews were offered to 100 % of those with a baseline K10 score of 25+, 75 % of those with a score 16–24, and one-sixth of those scoring 10–15. It was anticipated that this formula would result in interviews being completed by approximately equal numbers of participants in each of the psychological distress categories. WHM-CIDI-3.0 interviews were completed within two weeks of the K10 where possible, however participant preference and availability was also taken into consideration. The WMH-CIDI-3.0 has excellent inter-rater reliability, good validity and test-retest reliability, and is an acceptable method to determine lifetime psychiatric diagnoses [7], through both face-to-face and telephone delivery.

ARMHS participants completed the following WMH-CIDI-3.0 modules by telephone: PTSD, generalised anxiety disorder, social phobia, agoraphobia, panic attack and panic disorder (“anxiety disorder”); unipolar and bipolar major depression, dysthymia and minor depression (“affective disorder”); alcohol or drug abuse or dependence (“substance use disorder”); and suicidal ideation and attempts.

A shortened version of the WMH-CIDI-3.0 PTSD section was administered, enquiring about the lifetime occurrence of the PTEs presented in Table 1 (as opposed to the full PTSD section, which includes 28 separate PTEs). Respondents could endorse more than one event. For each event, the age of the first occurrence and the number of total occurrences were recorded. For the present study, PTSD diagnoses were determined based on symptoms experienced in association with each participant’s self-reported “worst” PTE.

**Table 1:** ARMHS participants who completed the WMH-CIDI-3.0 PTSD section (*N* = 623) – Lifetime PTSD by PTE

Potentially trauma event (PTE)	N (%)	Diagnosis of PTSD	Adjusted Odds Ratios (AOR) for lifetime PTSD *(p-value)*	
		N (%)	AOR1: Adjusted for age & gender	AOR2: Adjusted for age, gender & other PTEs
Combat	22 (3.5)	9 (40.9)	4.25 (.003)	1.80 (.274)
Life threatening accident	144 (23.1)	44 (30.6)	1.72 (.016)	1.06 (.836)
Natural disaster	66 (10.6)	23 (34.8)	2.11 (.009)	1.25 (.497)
Life threatening illness	155 (24.9)	45 (29.0)	1.93 (.004)	1.42 (.172)
Physically assaulted	161 (25.8)	69 (42.9)	3.78 (<.001)	2.53 (<.001)
Sexually assaulted	110 (17.7)	46 (42.2)	2.51 (<.001)	1.32 (.299)
Unexpected death of a loved one	305 (49.0)	107 (35.1)	3.37 (<.001)	2.84 (<.001)
Child had life threatening illness	112 (18.0)	38 (33.9)	1.88 (.007)	1.17 (.561)
Witnessed injury, death, dead body	208 (33.4)	75 (36.1)	3.15 (<.001)	1.88 (.007)
Other^a^	144 (23.1)	54 (37.2)	2.39 (<.001)	2.06 (.002)
Any event	514 (82.5)	151 (29.4)		

*WMH-CIDI-3.0* World Mental Health Composite International Diagnostic Interview, Version 3.0; *ICD-10* International Classification of Diseases, Version 10; reference category for AORs = participants completing the WMH-CIDI-3.0 who did not experience the focal trauma event

^a^Most common “other” events were: life-threatening event to family member, and death of a family member (not “unexpected” death); others included mental illness of another, divorce/separation, threat of abuse, and work-related trauma

#### *Demographics*

A self-report postal survey assessed a range of demographic characteristics, including age, gender, marital status and education.

#### *Current functioning*

Psychological distress was assessed using the Kessler-10, a 10-item measure of symptoms of distress during the previous four weeks; scores range from 10 to 50 with higher scores indicating greater distress [8]. Alcohol use was assessed by the Alcohol Use Disorder Identification Test [9], a 10-item measure of alcohol use during the previous six months, where higher scores represent higher alcohol use and/or related harmful behaviours. Self-reported use of mental health services in the previous 12 months was also recorded.

#### Recent adverse life events

Recent adverse life events were assessed using the List of Threatening Experiences [10], a 12-item measure of events in the past 12 months, including marital difficulties, becoming unemployed, and major financial difficulties.

#### *Personality variables*

Personal hopefulness was measured by the 12-item version of the Hunter Opinions and Personal Expectations Scale [11, 12]. Higher scores represent higher personal hopefulness. Neuroticism was assessed using the 12-item short form Eysenck Personality Inventory measure [13], from which a 7-item subset was identified (being easily hurt, a nervous person, a worrier, being highly strung, suffering from nerves, worrying too long, and often guilty) that conceptually reflected pre-dispositional or trait characteristics, and may be usefully delineated from current distress items [14]. Higher scores indicate higher neuroticism.

#### *Community factors*

##### Individual report

These included: a) living on a farm; b) proportion of life spent living in a rural area; and c) infrastructure and services accessibility, which assessed common concerns in rural communities on a 5-point Likert scale ranging from ‘not at all’ to ‘a lot’ (see Kelly et al. [14] for details).

##### Secondary data sources

Remoteness of residence was calculated using the Accessibility/Remoteness Index of Australia Plus [15], which was used to determine the Australian Standard Geographic Classification of areas. This method is used to allocate locations into five categories: major cities, inner regional, outer regional, remote and very remote.

##### Social support

Perceived availability of social support was assessed using the Interview Schedule for Social Interactions – Availability of Attachment Scale [16].

#### *Statistical methods*

Diagnoses of psychiatric disorder, including PTSD, were calculated according to the WMH-CIDI diagnostic algorithms using SAS 9.2 (SAS Institute Inc., Cary, North Carolina). Data were analysed using SPSS (version 20; SPSS, Chicago, IL, USA). Logistic regressions were used to examine the overall likelihood of lifetime ICD-10 PTSD among those who reported each lifetime PTE. This was initially done controlling for age category and gender (which are likely to impact on rates of exposure, and the timing and reporting for some PTEs), followed by an additional adjustment for the effects of any other lifetime PTEs. Results are reported as adjusted odds ratios (AOR) with *p*-values. The threshold for statistical significance was set at *p* < .01 to partially adjust for multiple tests.

We also examined social, psychological and demographic differences across four sub-groups: adults with lifetime PTEs and either current PTSD (i.e., symptoms in the last 12 months; *Group A*) or past PTSD (without 12-month symptoms; *Group B*); lifetime PTE without meeting criteria for lifetime PTSD (*Group C*); and those with no history of PTEs (*Group D*). Continuous variables were analysed using one-way analysis of covariance (ANCOVA), while for categorical variables, chi-square analyses were used; once again, age category and gender were included as covariates.

## Results

**Fig. 1:**
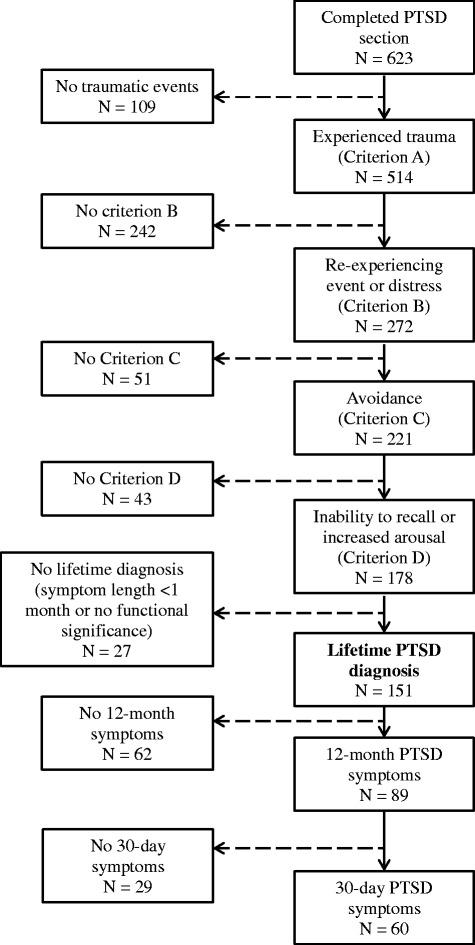
Flow chart of baseline ARMHS participants who completed the PTSD section of the WMH-CIDI-3.0

Among the 623 ARMHS participants who completed the PTSD section of the WMH-CIDI-3.0, 376 (60.4 %) were female, 420 (67.4 %) had completed secondary school, and 570 (91.5 %) had been married or in a *de facto* relationship either past or present. The mean age of the sample was 55.5 years (SD 13.9). Overall, 514 (82.5 %) participants reported at least one lifetime PTE, and 151 (24.2 % of the total sample) met criteria for lifetime PTSD (or 16.0% after backweighting for stratification). The diagnostic process for participants with lifetime PTSD is depicted in Fig. 1.

### Occurrence of PTEs and PTSD

Table 1 describes the frequency of PTEs and the associated lifetime rates of PTSD (potentially from any trauma exposure). Most participants (514/623, or 82.5 %) reported at least one PTE, the most frequent being: the unexpected death of a loved one; witnessing an injury, death or a dead body; physical assault; and having a life-threatening illness. Adjusting for age category and gender (AOR1 models), all PTEs (except life threatening accident, *p* = .016) were significantly associated with a higher likelihood of lifetime PTSD. As an individual may have experienced more than one PTE, additional adjustments were made for the presence of other PTEs (AOR2 models). Developing PTSD (adjusted for the presence of other endorsed events) was significantly associated with having experienced the unexpected death of a loved one, physical assault, and, to a lesser extent, with ‘other’ PTEs and witnessing injury or death.

### Factors associated with lifetime PTEs and PTSD

Table 2 reports comparisons between sub-groups categorized on the basis of their traumatic experience and PTSD history, to examine the factors associated with lifetime occurrence of PTSD, and those factors that may be “protective” in the event of PTE exposure. Current distress was significantly higher in those with any PTSD than in those with either no PTE or PTE without PTSD.

**Table 2:** Sub-group comparisons among ARMHS participants who completed a WMH-CIDI-3.0 – based on Potentially Traumatic Event (PTE) experience and Post-Traumatic Stress Disorder (PTSD) history

Characteristic	A	B	C	D	Overall analysis^a^ *p-value*	Pattern of sub-group differences (post-hoc tests)
	Trauma + current PTSD	Trauma + past PTSD	Trauma without PTSD	No trauma		
	(*N* = 89)	(*N* = 62)	(*N* = 363)	(*N* = 109)		
	Mean (SD)	Mean (SD)	Mean (SD)	Mean (SD)		
Demographics						
Age	51.9 (13.1)	53.7 (12.0)	57.2 (13.5)	54.1 (16.0)	.004	A < C
Gender (male), % (n)	32.6 (29)	29.0 (18)	45.2 (164)	33.0 (36)	.010	nil
Ever married, % (n)	93.3 (83)	83.3 (50)	93.4 (338)	90.8 (95)	.061	
Completed high school, % (n)	69.7 (62)	58.1 (36)	69.1 (251)	65.1 (71)	.331	
Current functioning						
Current psychological distress	23.1 (7.8)	20.7 (6.1)	18.2 (6.1)	17.2 (6.2)	<.001	A,B > C,D
Alcohol use	5.4 (6.8)	3.4 (4.7)	4.1 (4.1)	3.7 (3.5)	.034	
Service use for mental health problem, % (n)	61.8 (47)	44.4 (24)	31.0 (95)	23.4 (22)	<.001	A > C,D; B > D
Adverse life events	2.7 (2.0)	2.3 (1.9)	1.9 (1.7)	1.1 (1.2)	<.001	A > C > D; B > D
Dispositional variables						
HOPES-12^◊^	2.5 (0.7)	2.7 (0.6)	2.8 (0.6)	2.8 (0.6)	.017	
Neuroticism	3.7 (2.0)	3.2 (2.1)	2.5 (2.1)	2.8 (2.1)	<.001	A > C,D
Community factors						
Live on farm, % (n)	23.6 (21)	23.3 (14)	25.4 (91)	18.5 (20)	.534	
Proportion of life in a rural area	0.7 (0.3)	0.7 (0.3)	0.7 (0.3)	0.7 (0.3)	.876	
Infrastructure and services accessibility	2.9 (1.0)	2.8 (1.0)	2.5 (1.0)	2.5 (1.0)	.001	A > C,D
Remoteness category, % (n)						
Inner regional	35.5 (22)	40.4 (36)	41.3 (150)	36.7 (40)	.132	
Outer regional	45.2 (28)	41.6 (37)	36.1 (131)	34.9 (38)		
Remote	6.5 (4)	9.0 (8)	14.9 (54)	22.0 (24)		
Very remote	12.9 (8)	9.0 (8)	7.7 (28)	6.4 (7)		
Social support	4.2 (2.0)	4.5 (1.7)	5.0 (1.6)	5.3 (1.2)	<.001	A < C,D; B < D
Other psychiatric morbidity						
Lifetime suicidal ideation, % (n)	40.4 (36)	37.1 (23)	22.0 (80)	13.8 (15)	<.001	A > C,D; B > D
Lifetime affective disorder, % (n)	42.7 (38)	32.3 (20)	16.8 (61)	18.3 (20)	<.001	A > C,D
Lifetime anxiety disorder, % (n)	100 (89)	100 (62)	25.3 (92)	17.4 (19)	<.001	A > C,D; B > D
Lifetime substance use disorder, % (n)	40.4 (36)	32.3 (20)	24.0 (87)	11.9 (13)	<.001	A > C > D; B > D
PTE history						
Age at first trauma	17.4 (14.1)	18.5 (12.5)	21.4 (15.5)	N/A	.045	
Number of traumatic events	3.4 (1.7)	3.3 (1.9)	2.5 (1.5)	N/A	<.001	A,B > C

K10: Kessler 10; AUDIT: Alcohol Use Disorder Identification Test; HOPES-12: Hunter Opinions and Personal Expectations Scale-12 (^◊^based on a subset of 384 participants); WMH-CIDI-3.0: World Mental Health Composite International Diagnostic Interview Version 3.0

^a^Overall comparisons (chi-square tests or ANCOVAs, controlling for age category and gender), with post-hoc follow-up tests

The number of recent adverse life events was highest in those with a lifetime diagnosis of PTSD (particularly those with current symptoms), and lowest in those with no PTEs. Events listed in the Life Events Scale included interpersonal events (such as household arguments or difficulties with partner), alongside events that overlap with PTSD items. Further analysis indicated that the association between current PTSD and recent adverse life events was not accounted for by these PTEs occurring in the past 12 months in this group.

With regards to personal vulnerability factors, people with current PTSD reported lowest levels of hopefulness and greater neuroticism when compared to those who reported either PTE but no PTSD, or no PTE. There was no influence of putative measures of exposure to rural environments (e.g., duration in rural area) or rural specific characteristics (e.g., living on a farm). Those with current PTSD also reported significantly greater concerns about infrastructure and services accessibility in rural communities, potentially reflective of their current need for mental health services.

Participants with current PTSD reported significantly higher rates of lifetime suicidal ideation, lifetime affective disorder and lifetime substance use than those without PTSD (Groups C and D). In addition, those with past PTSD reported significantly higher rates of lifetime suicidal ideation, and lifetime substance use than those with no reported trauma (Group D); the lifetime anxiety disorder profiles in Table 2 are consistent with the sub-group defining characteristics. The number of traumatic events was also significantly higher in those with current or past PTSD compared to those who experienced trauma and did not develop PTSD.

## Discussion

The majority of our sample reported experiencing at least one lifetime PTE, which is broadly consistent with findings from other Australian [17, 18] and international studies [19, 20], although rates vary with trauma definitions and sampling methods [21]. In addition, one-quarter of our rural sample reported lifetime PTSD; these rates are higher than the comparable 2007 Australian National Survey of Mental Health and Wellbeing rates (12.2 %) [22], although our sample was older and the ARMHS baseline response rates were also lower than in the National Survey [6, 23]. Physical and sexual assault were associated with the highest raw rates of PTSD, which is consistent with past studies [20].

The majority of respondents who experienced a PTE but did not meet diagnostic criteria for PTSD were excluded at Criterion B (re-experiencing the event, or experiencing distress when reminded of the event). This was reflected in the analysis in Table 2; participants who had no diagnosis of PTSD were similar to those who had not experienced a PTE in terms of their current functioning and wellbeing, indicating that the events may have had limited long-term psychological and social impact. In contrast, those who had experienced PTSD, either in the past or currently, reported significantly poorer scores on a range of domains than those with no PTSD history. In particular, those with current PTSD reported the highest levels of psychological distress and mental health service use, while they were also more likely to report concerns about service availability, and had the poorest perceived social support.

Both current and past PTSD were associated with higher rates of psychological comorbidities, with these groups more likely to meet diagnostic criteria for lifetime affective and substance use disorders, and suicidal ideation. While it is possible that these additional diagnoses are responsible for the higher reliance on mental health services among these participants, we were not able to determine this in the present analysis.

There was some evidence for the role of personal vulnerability in the progression to PTSD, with higher neuroticism reported by those with current PTSD compared to those with no PTSD history. This may be indicative of the contribution of trait as well as state factors in PTE exposure and outcomes, or may suggest that neuroticism predisposes to more chronic and persistent symptoms following a PTE. On average, respondents who developed lifetime PTSD experienced a greater number of PTEs than those who did not, implying a cumulative effect of PTEs on negative long-term outcomes. Interestingly, we did not find any differences in PTE exposure by remoteness or duration of residence in a rural area, which contrasts with previous research suggesting that rural characteristics may increase the likelihood of PTEs or PTSD [1, 2, 4].

Limitations of this study include the use of lifetime recall to determine patterns of psychiatric disorder, which is subject to previously identified inaccuracies, with people less likely to meet criteria as time since the episode increases [24]. The raw rates of PTE are not reflective of population rates, given the methods through which this sample was selected to undertake the WMH-CIDI-3.0. In addition, PTSD estimates were based on self-reported “worst” experience, which is also subject to possible recall and other biases.

Beyond the above findings, this study’s main contribution is in the investigation of characteristics of participants according to PTE exposure and the development of PTSD. Our findings support previous urban-based research indicating an important role for social cohesion in protecting against the development of PTSD following a PTE, after controlling for factors such as number of trauma events and socio-demographic characteristics [25]. While Johns et al. [25] found evidence that social support influences the psychological response to a PTE (hence lower support increases the likelihood of PTSD), previous research also shows that interpersonal conflict and declines in social support may occur as a consequence of PTEs [26]. The cross-sectional nature of our analysis, and our use of essentially lifetime measures of PTEs and PTSD, prevented us from exploring the direction of this relationship further. Social support has consistently been observed as a particularly important construct in rural areas, contributing significantly to better mental health [27] and overall wellbeing [28], as well as protecting against psychological distress [29] and the development of suicidal ideation [30]. This accords with evidence regarding the protective role of social support in the setting of trauma and adversity [25]. Promoting existing support networks within rural communities may be an important strategy to enhance recovery among those exposed to PTEs. A focus of a number of existing programs (such as the Department of Veterans’ Affairs *“Health policy for the veteran community in rural and remote areas”* and Cancer Australia’s *“Supporting women in rural areas diagnosed with breast cancer”* program) is to utilise existing community structures to provide support to individuals in the event of personal adversity. The findings indicate the significant effects of PTSD on multiple domains of current functioning among rural residents. This is of concern given the context of limited accessibility and limited range of health services in rural areas, significant delays in treatment seeking [31] and the resulting potential for greater unmet treatment need and disability associated with PTSD.

## Conclusions

Ultimately, our findings show that the effects of a PTE, particularly one that is perceived as distressing at the time that it occurs, are far-reaching and may impact on multiple domains of an individual’s life for many years after its occurrence. As predicted, those with a PTE history, and particularly those with current PTSD, were impaired in multiple areas of their functioning and wellbeing. Our results suggest that the immediate response to a PTE may be of particular importance. Hence, increasing the availability of services for rural residents in the period directly following a PTE may be a useful strategy to prevent the development of PTSD and to assist with long-term recovery from the PTE.
